# Study of the Pithecanthropus Erectus, or Ape-Man1Read before the Chicago Academy of Medicine, June 27, 1904.

**Published:** 1905-10

**Authors:** Eugene S. Talbot


### STUDY OF THE PITHECANTHROPUS ERECTUS, OR APE-MAN.[1]

BY EUGENE S. TALBOT, M.S., D.D.S., M.D., LL.D.

Haeckel suggested many years ago that in a hypothetic continent called Sumeria, of which Java, Borneo, etc., were the remains, as having a continual fauna, man originated. From time to time pithecoid types of skull, like the Neanderthal and Spy type, were discovered in Europe, albeit comparatively late in geologic history in the pleistocene rather than the tertiary (eocene, miocene, and phocene). The face type was pithecoid, but the skull, while low in cranial capacity, was far above the highest anthropoid ape. Evidences of man in the shape of cut whalebones had been found in the pleocene to the Neanderthal and Spy type of man, despite the fact that his ape-like face and femur was not the oldest type.

In 1892 there was much excitement in the biologic world over the discovery by Dr. Dubois, in Java, of a few bones and teeth at a distance of thirty-two feet six inches below the surface of the ground upon a river bank. Great stress was laid upon the antiquity of these fossils, because of the distance below ground, the character of soil, etc., but Dubois believed them to be pleistocene. "The first sight of the fossils was a surprise, as they were evidently much older than appeared from Dubois's description. All the physical characters impressed Marsh with the idea that the remains were pliocene and not pleistocene. The discoveries consisted of a tooth found first in the bank of the river thirty-nine inches below the water-level during the dry season. A month later the skull was found a short distance from the tooth; two months later another tooth was found ten feet from where the skull was located; still later a femur was discovered. These bones were

[1] Read before the Chicago Academy of Medicine, June 27, 1904.

carefully studied by Dubois, who believed them to belong to a new family which he named the pithecanthropidæ, distinguished mainly by the following characteristics.

The brain cavity is absolutely larger and, in proportion to the size of the body, much more capacious than in the Simiadæ, yet less so than in the Hominidæ. Cranial capacity about two-thirds the human average. Inclination of the nuchal surface of the occiput considerably greater than in the Simiadæ. Dentition, although retrogressive, still the human type. Femur equal in its dimensions to that of man, and like that adapted for walking in an upright position, unlike the Spy femur here, which was more pithecoid.

Of this skull the upper portion alone is preserved, the line of fracture extending from the glabella backward irregularly to the occiput, which it divides somewhat below the upper nuchal line. The cranium seen from above is an elongated oval in outline, dolichocephalic, and is distinguished from that of other anthropoid apes by its large size and its higher arching in the coronal region. The greatest length from the glabella to the posterior projection of the occiput is 185 millimetres; the greatest breadth is 130 millimetres; and the smallest behind the orbit is 90 millimetres. The cranium in its original condition must have been of somewhat larger dimensions. The upper surface of the skull is without ridges, and the sutures all appear to be obliterated.

This dolichocephalic skull, with an index of seventy degrees, is readily distinguished from the brachycephalic orang-outang skull. The absence of the characteristic cranial crests separate it from the dolichocephalic skull of the adult gorilla. In its smooth upper surface and general form it resembles somewhat the chimpanzee skull, and still more that of the gibbon (Hylobates).

In this hurried communication I had no means of obtaining a more complete study of the cast. There are, however, two or three points of interest which seem to indicate that this skull belongs to a type neither man nor ape. The first is the peculiar length and shape of the orbital ridges. If the animal had walked upon all fours, such prominent ridges would have been unnecessary for the protection of the eyes from the sun and violence of all kinds. They are much more prominent and thinner than those of the Neanderthal, Engis, or Spy skulls. If he had walked continually upon all fours, the occipital protuberance and the superior curved line for the attachment of strong muscles would have been excessively developed. In the place of occipital protuberance appears a depression.

The tooth, of which I have only a picture, is the last upper molar of the right side in good preservation. It indicates an adult. The crown is subtriangular in form, with the corners rounded and the narrowest portion behind. The antero-posterior diameter of the crown in 11.3 millimetres and the transverse 15.3 millimetres. The grinding surface of the crown is concave and less rugose than in existing anthropoid apes. The diverging roots are a pithecoid feature.

How Dubois decided that the tooth was a right superior third molar, or even a third molar at all, is a mystery to me. The picture (a drawing) shows a very small crown as compared with the roots. The roots are short and strong. The third molars in human beings usually have their roots close together, crowns small, with long, thin enamel, flat grinding surface. The short crown, heavy enamel, concave grinding surface, a few rugose, short, thick, heavy, diverging roots all indicate a lower type than those of man.

